# Cardioprotective role of leaves extracts of *Carissa opaca* against CCl_4_ induced toxicity in rats

**DOI:** 10.1186/1756-0500-7-224

**Published:** 2014-04-09

**Authors:** Sumaira Sahreen, Muhammad Rashid Khan, Rahmat Ali Khan, Huda Mohammed Alkreathy

**Affiliations:** 1 Botanical Sciences Division, Pakistan Museum of Natural History, Islamabad, Pakistan; 2 Department of Biochemistry, Faculty of Biological Sciences, Quaid-i-Azam University, Islamabad, Pakistan; 3 Department of Biotechnology, Faculty of Sciences, University of Science and Technology, Bannu, Pakistan; 4 Department of Pharmacology, Faculty of Medicine, King Abdulaziz University, Jeddah, Saudi Arabia

**Keywords:** DNA damages, Lipid peroxidation, Histopathalogical, CCl_4_

## Abstract

**Background:**

*Carissa opaca* are used traditionally in Pakistan for the treatment of various human ailments. Therefore, the study is arranged out to assess the cardio protective potential of different fractions of *Carissa opaca* leaves on CCl_4_-induced oxidative trauma in kidney.

**Methods:**

The parameters studied in this respect were the cardiac function test (CK (U/l), CKMB (U/l), genotoxicity (% DNA fragmentation), characteristic morphological findings and antioxidant enzymatic level of cardiac tissue homogenate.

**Result:**

The protective effects of various fractions of *Carissa opaca* (*C. opaca*) leaves extract against CCl_4_ administration was reviewed by rat cardiac functions alterations. Chronic toxicity caused by eight week treatment of CCl_4_ to the rats significantly changed the cardiac function test, decreased the activities of antioxidant enzymes and glutathione contents whereas significant increase was found in lipid peroxidation comparative to control group. Administration of various fractions of *C. opaca* leaves extract with CCl_4_ showed protective ability against CCl_4_ intoxication by restoring the cardiac functions alterations, activities of antioxidant enzymes and lipid peroxidation in rat. CCl_4_ induction in rats also caused DNA fragmentation and histopathalogical abnormalities which were restored by co-admistration of various fraction of *C. opaca* leaves extract.

**Conclusion:**

Results revealed that various fraction of *C. opaca* are helpful in cardiac dysfunctions.

## Background

Plants are used to extract pure compounds and development of new drugs, has supreme frankness of chemical diversity. Natural products derived from plant extracts/fractions are novel therapeutic agents for various infectious as well as degenerative diseases [1]. People of developed countries have turned back their attention towards botanicals as medical care, but countries like Pakistan, China and India seek help from botanical healers since centuries to till date because they grant them substitutive health care services based on botanicals as an accessible and economical source in comparison to synthetic medicines [2]. Hence, medicinal plants are considered as therapeutic agents against various diseases. No doubt indigenous use of plants is unlimited, but it is necessary to discover the pharmaceutically important agents responsible for protection against lethal diseases. It is anticipated that about one quarter of approved modern medicines has been derived from botanicals [3]. Several anti-cancer drugs including, vinblastine and paclitaxel are exclusively derived from botanicals. Similarly, aspirin, a recognized pain killer, was actually a derivative of *Salix* and *Spiraea* species [4]. Regarding the excessive use of botanicals as health care medicines, it has become an important step to screen the medicinal plants for bioactive compounds as a source of new antibiotic and cancer-related drugs. *Carissa opaca* Stapf ex Hanes is a 2–3 meter tall evergreen shrub containing glabrous or puberulous branches with opposite and ovate glabrous leaves, hard and sharp spines arising between the petiole. This plant is also reported in some areas of India, Burma and Sri Lanka [5]. Jabeen *et al*. [6] reported the use of plant against worm infested sores of animals, asthma, stimulants and fly repellent. According to Saghir *et al*. [7] fruits and leaves are cardiac and stimulant. Abbasi *et al*. [8] described the traditional use of plant for the treatment of hepatitis and jaundice. Researchers have reported that plant is purgative, and give relief from cough, diarrhea and fever [9]. Ahmad *et al*. [10] described the ethnobotanical use of stems, leaves and fruits of *C. opaca* in eye disorders and reported that mixture of *C. opaca* with roots of M*imosa pudica* is used as aphrodisiac. Carbon tetrachloride (CCl_4_), a clear, colorless and nonflammable synthetic liquid, is a renowned model compound for producing chemical tissue toxicity by creation of free radicals in liver, kidney, heart, lung, testis, brain and blood [11,12]. It is bio transformed by hepatic microsomal cytochrome P450 to trichloromethyl-free radical (CCl_3_ or CCl_3_OO) [13], which in turn, instigate lipid peroxidation process [14,15]. The most widely established means of CCl_4_ induced cardiotoxicity is the creation of free radicals which is a rate limiting process in tissue peroxidative damage [16]. The present study was conducted to examine the toxic upshots of CCl_4_ plus to compare the beneficial effects of plant extracts on heart tissue of various experimental groups.

## Methods

### Plant collection

*C. opaca* leaves were collected in June 2011 from the Quaid-i-Azam University Islamabad, Pakistan. The plants were recognized by their local names and then validated by Dr. Mir Ajab Khan, Department of Plant Sciences, Quaid-i-Azam University, Islamabad. A voucher specimen with Accession No. 24561 (*C. opaca*) was deposited at the Herbarium of Pakistan Quaid-i-Azam University, Islamabad Pakistan.

### Extract preparation

The collected plant samples were cleaned to get rid of dust particles and then dried under shade for one to two weeks. Willy Mill of 60-mesh size was used to prepare powder of dried samples and then each powdered plant sample was used for further solvent extraction. First of all, 5 kg of powdered sample was extracted twice with 10 L of 95% methanol at 25°C for 48 h. For filtration Whatman No. 1 filter paper was used and then filtrate was concentrated on rotary evaporator (Panchun Scientific Co., Kaohsiung, Taiwan) under reduced pressure at 40°C. In order to resolve the compounds with escalating polarity, a part of the extract was suspended in distilled water and subjected to liquid-liquid partition by using solvents in a sequence of n-hexane, ethyl acetate and methanol. After fractioning, the solvent of respective fractions was also evaporated by rotary evaporator. Extract was dried and then stored at 4°C for further *in vivo* investigation.

### Experimental plan

Six-week-old male Sprague Dawley rats weighing 180 ± 10 g were provided with food and water *ad libitum* and kept at 20–22°C on a 12-h light–dark cycle. All experimental procedures involving animals were conducted in accordance with the guidelines of National Institutes of Health (NIH guidelines). The study protocols were approved by Ethical committee of Quaid-i-Azam University Islamabad. The rats were acclimatized to laboratory condition for 7 days before commencement of experiment. For chronic toxicity eight week experiment was designed. 42 male albino rats were randomly divided into seven groups (6 rats of each group). Administration of CCl_4_ (0.5 ml/kg b.w., 20% CCl_4_/olive oil) was intraperitoneally (i.p.) twice a week for eight weeks. At the same time, the rats were administered individually silymarin (50 mg/kg b.w.) and extract (200 mg/kg b.w.) orally twice a week for eight weeks.

### Experimental protocol

Following dosing plan was adapted for the study.

Group I. the normal control received only feed

Group II. Olive oil (0.5 ml/kg b.w. i.p.) + DMSO (0.5 ml/kg b.w. orally)

Group III. CCl_4_ twice a week (0.5 ml/kg b.w. i.p., 20% CCl_4_/olive oil)

Group IV. CCl_4_ twice a week (0.5 ml/kg b.w. i.p.) + sylimarin (50 mg/kg b.w. orally)

Group V. CCl_4_ twice a week (0.5 ml/kg b.w. i.p.) + HLC (200 mg/kg b.w., orally)

Group VI. CCl_4_ twice a week (0.5 ml/kg b.w. i.p.) + ELC (200 mg/kg b.w., orally)

Group VII. CCl_4_ twice a week (0.5 ml/kg b.w. i.p.) + MLC (200 mg/kg b.w., orally)

At the end of eight weeks, after 24 h of the last treatment, Urine was collected and stored at −70°C for further analysis, and then animals were given chloroform anesthesia and dissected from ventral side. Blood was drawn prior to the excision of tissues. The serum was separated and stored at −80°C after separation until it was assayed as described below. After taking blood the heart was removed and washed in ice cold saline. Subsequently, half of the organs were treated with liquid nitrogen and stored at −80°C for further enzymatic and DNA damage analysis while the other portion was processed for histology.

### Biochemical investigations

In order to evaluate the pharmacological effects of different fractions of *C. opaca* leaves extract against the toxicity induced with CCl_4_ in rats following assays had been carried out.

### Biochemical analysis of serum

Estimation of serum marker enzymes viz; CK, CKMB was carried out by using standard AMP diagnostic kits.

### Assessment of antioxidant enzymes

100 mg of heart tissue was homogenized in 10 volume of 100 mM KH2PO4 buffer containing 1 mM EDTA, pH 7.4 and centrifuged at 12,000 × g for 30 min at 4°C. The supernatant was collected and used for the following experiments.

### Catalase assay (CAT)

CAT activity was determined by the modified protocol of Khan *et al.*[17]. The reaction solution of CAT activities contained: 2.5 ml of 50 mM phosphate buffer (pH 5.0), 0.4 ml of 5.9 mM H_2_O_2_ and 0.1 ml enzyme extract. Changes in absorbance of the reaction solution at 240 nm were determined after one minute. One unit of CAT activity was defined as an absorbance change of 0.01 as units/min.

### Peroxidase assay (POD)

Activities of POD were determined by the modified protocol of Khan *et al.*[17]. The POD reaction solution contained: 2.5 ml of 50 mM phosphate buffer (pH 5.0), 0.1 ml of 20 mM guaiacol, 0.3 ml of 40 mM H_2_O_2_ and 0.1 ml enzyme extract. Changes in absorbance of the reaction solution at 470 nm were determined after one minute. One unit of POD activity was defined as an absorbance change of 0.01 units/min.

### Superoxide dismutase assay (SOD)

SOD activity was estimated by the method of Kakkar *et al*. [18]. Reaction mixture of this method contained: 0.1 ml of phenazine methosulphate (186 μM), 1.2 ml of sodium pyrophosphate buffer (0.052 mM; pH 7.0), 0.3 ml of supernatant after centrifugation (1500 × g for 10 min followed by 10000 × g for 15 min) of heart homogenate was added to the reaction mixture. Enzyme reaction was initiated by adding 0.2 ml of NADH (780 μM) and stopped after 1 min by adding 1 ml of glacial acetic acid. Amount of chromogen formed was measured by recording color intensity at 560 nm. Results are expressed in units/mg protein.

### Glutathione-S-transferase assay (GST)

Glutathione-S-transferase activity was assayed by the method of Habig *et al*. [19]. The reaction mixture consisted of 1.475 ml phosphate buffer (0.1 mol, pH 6.5), 0.2 ml reduced glutathione (1 mM), 0.025 ml (CDNB) (1 mM) and 0.3 ml of homogenate in a total volume of 2.0 ml. The changes in the absorbance were recorded at 340 nm and enzymes activity was calculated as nM CDNB conjugate formed/min/mg protein using a molar extinction coefficient of 9.6 × 10^3^ M^−1^ cm^−1^.

### Glutathione reductase assay (GR)

Glutathione reductase activity was determined by method of Carlberg and Mannervik [20]. The reaction mixture consisted of 1.65 ml phosphate buffer: (0.1 mol; pH 7.6), 0.1 ml EDTA (0.5 mM), 0.05 ml oxidized glutathione (1 mM), 0.1 ml NADPH (0.1 mmol) and 0.1 ml of homogenate in a total volume of 2 ml. Enzyme activity was quantitated at 25°C by measuring disappearance of NADPH at 340 nm and was calculated as nM NADPH oxidized/min/mg protein using molar extinction coefficient of 6.22 × 10^3^ M^−1^ cm^−1^.

### Glutathione peroxidase assay (GPx)

Glutathione peroxidase activity was assayed by the modified method of Khan *et al*. [21]. The reaction mixture consisted of 1.49 ml phosphate buffer (0.1 M; pH 7.4), 0.1 ml EDTA (1 mM), 0.1 ml sodium azide (1 mM), 0.05 ml glutathione reductase (1 IU/ml), 0.05 ml GSH (1 mM), 0.1 ml NADPH (0.2 mM), 0.01 ml H_2_O_2_ (0.25 mM) and 0.1 ml of homogenate in a total volume of 2 ml. The disappearance of NADPH at 340 nm was recorded at 25°C. Enzyme activity was calculated as nM NADPH oxidized/min/mg protein using molar extinction coefficient of 6.22 × 10^3^ M^−1^ cm^−1^.

### Quinone reductase assay (QR)

The activity of quinone reductase was determined by the method of Benson *et al*. [22]. The 3.0 ml reaction mixture consisted of 2.13 ml Tris–HCl buffer (25 mM; pH 7.4), 0.7 ml BSA, 0.1 ml FAD, 0.02 ml NADPH (0.1 mM), and 0.l ml of homogenate. The reduction of dichlorophenolindophenol (DCPIP) was recorded at 600 nm and enzyme activity was calculated as nM of DCPIP reduced/min/mg protein using molar extinction coefficient of 2.1 × 10^4^ M^−1^ cm^−1^.

### Reduced glutathione assay (GSH)

Reduced glutathione was estimated by the method of Jollow *et al*. [23]. 1.0 ml sample of homogenate was precipitated with 1.0 ml of (4%) sulfosalicylic acid. The samples were kept at 4°C for 1 h and then centrifuged at 1200 × g for 20 min at 4°C. The total volume of 3.0 ml assay mixture contained 0.1 ml filtered aliquot, 2.7 ml phosphate buffer (0.1 M; pH 7.4) and 0.2 ml DTNB (100 mM). The yellow color developed was read immediately at 412 nm on a SmartSpecTM plus Spectrophotometer. It was expressed as μM GSH/g tissue.

### Estimation of lipid peroxidation assay

The assay for lipid peroxidation was carried out following the modified method of Iqbal *et al*. [24]. The reaction mixture in a total volume of 1.0 ml contained 0.58 ml phosphate buffer (0.1 M; pH 7.4), 0.2 ml homogenate sample, 0.2 ml ascorbic acid (100 mM), and 0.02 ml ferric chloride (100 mM). The reaction mixture was incubated at 37°C in a shaking water bath for 1 h. The reaction was stopped by addition of 1.0 ml 10% trichloroacetic acid. Following addition of 1.0 ml 0.67% thiobarbituric acid, all the tubes were placed in boiling water bath for 20 min and then shifted to crushed ice-bath before centrifuging at 2500 × g for 10 min. The amount of TBARS formed in each of the samples was assessed by measuring optical density of the supernatant at 535 nm using spectrophotometer against a reagent blank. The results were expressed as nM TBARS/min/mg tissue at 37°C using molar extinction coefficient of 1.56 × 10^5^ M^−1^ cm^−1^.

### Hydrogen peroxide assay (H_2_O_2_)

Hydrogen peroxide (H_2_O_2_) was assayed by H_2_O_2_-mediated horseradish peroxidase-dependent oxidation of phenol red by the method of Pick and Keisari [25]. 2.0 ml of homogenate sample was suspended in 1.0 ml of solution containing phenol red (0.28 nM), horse radish peroxidase (8.5 units), dextrose (5.5 nM) and phosphate buffer (0.05 M; pH 7.0) and were incubated at 37°C for 60 min. The reaction was stopped by the addition of 0.01 ml of NaOH (10 N) and then centrifuged at 800 × g for 5 min. The absorbance of the supernatant was recorded at 610 nm against a reagent blank. The quantity of H_2_O_2_ produced was expressed as nM H_2_O_2_/min/mg tissue based on the standard curve of H_2_O_2_ oxidized phenol red.

### Molecular studies

DNA had been isolated and its fragmentation percent was quantified in molecular studies of *in vivo* toxicity.

### DNA fragmentation assay with diphenylamine reaction

DNA fragmentation from tissue extract was determined using the procedure of Wu *et al*. [26]. 100 mg tissue was homogenized in TTE solution. 0.1 ml of homogenate was labeled B, centrifuged at 200 × g at 4°C for 10 min, got supernatant labeled S. S tubes were centrifuged at 20,000 × g for 10 min at 4°C to separate intact chromatin, was labeled T. 1.0 ml of 25% TCA was added in all tubes T, B, S and incubated over night at 4°C. After incubation precipitated DNA was recovered by pelleting for 10 min at 18,000 × g at 4°C. 160 μl of 5% TCA was added to each pellet and heated for 15 min at 90°C then 320 μl of freshly prepared DPA solution was added, vortexed and incubated for 4 hr 37°C. Optical density was read at 600 nm with a spectrophotometer (Smart spec ^TM^ Plus, catalog # 170–2525).

### DNA Isolations and ladder assay

DNA was isolated by using the methods of Wu *et al*. [26]. 100 mg of tissue in a petri dish was washed with DNA Buffer and homogenized in 1 ml lysis buffer. 100 μl of proteinase K (10 mg/ml) and 240 μl 10% SDS, shaked gently, and incubate overnight at 45°C in a water bath then 0.4 ml of phenol, was added shaked for 5–10 min, and centrifuge at 3000 rpm for 5 min at 10°C. Supernatant was mixed with 1.2 ml phenol, 1.2 ml Chloroform/isoamyl alcohol (24:1); shaked for 5–10 min, and centrifuged at 3000 rpm for 5 min at 10°C. 25 μl of 3 M sodium acetate (pH 5.2) and 5 ml ethanol was added with supernatant, shake until DNA was precipitated. DNA was washed with 70% ethanol, dried, dissolved in TE buffer and its concentration checked at 260 and 280 nm.5 μg of total DNA and 0.5 μg DNA standard per well were loaded on 1.5% agarose gel containing ethidium bromide. Electrophoresis was performed for 45 min with 100 V batteries, and DNA was observed under digital gel doc system and photographed.

### Histopathological study of tissue

After weighting the portion specifies for histology small pieces of tissue was fixed for 3–4 h in fixative sera followed by dehydration with ascending grades of alcohol (80%, 90%, and 100%) and transferred in cedar wood oil. When tissue becomes clear then all tissues were embedded in paraplast and prepared blocks for further microtomy. 3–4 μm thin slides were prepared with microtome; wax was removed, stained with hemotoxilin-eosin and photographed under light microscope at 10x and 40x.

### Statistical analysis

To find the different treatment effects of *in vivo* studies one way analysis of variance was carried by computer software SPSS 13.0. Level of significance among the various treatments was determined by LSD at 0.05% level of probability.

## Results

### Effects of *C. opaca* leaves against CCl_4_ induced cardio toxicity in rat

In order to evaluate the protective of different fractions of *C. opaca* leaves against CCl_4_-induced cardiac injuries, the level of antioxidant enzymes, an indicator of oxidative damage, was monitored. Cardiac function test and histology of the organ was also inspected to estimate the effects of different fractions of *C. opaca* leaves.

### Effects of *C. opaca* leave on cardiac function tests of rats

Table 1 summarizes the CK and CKMB level of serum. CCl_4_ intoxication markedly raised the content of CK and CKMB versus the control group. Different fractions of *C. opaca* leaves ameliorated the toxic effects of CCl_4_ and reversed towards the normal level. Alternatively, non-significant difference was noted in case of fractions alone for the above parameter as compare to the control group.

**Table 1:** **Effects of various fractions of *C. opaca *** **leaves on heart function tests**

**Group**	**CK (U/l)**	**CKMB (U/l)**
Control	94.36 ± 3.48e	118.97 ± 5.42d
Oil + DMSO	100.13 ± 5.44e	111.41 ± 3.09d
CCl_4_	910.46 ± 9.66a	259.23 ± 3.55a
Sily + CCl_4_	295.78 ± 8.37d	156.28 ± 3.00c
HLC + CCl_4_	630.66 ± 9.56b	239.68 ± 3.37b
ELC + CCl_4_	398.10 ± 8.29c	190.01 ± 4.17c
MLC + CCl_4_	311.66 ± 9.09d	158.68 ± 4.40c

Values are Mean ± SD (06 number). Sily = Silymarin.

a-g (Means with different letters) indicate significance at p < 0.05.

### Effects of *C. opaca* leave on cardiac enzymatic antioxidant levels

Alteration in tissue soluble protein and antioxidant defense system such as CAT, POD, SOD, TBARS and H_2_O_2_ is explained in Table 2. CCl_4_ intoxication markedly lessened the tissue protein and decreased the antioxidative status of cardiac catalase, peroxidase and superoxide dismutase while increased the lipid peroxidation and hydrogen peroxide levels in comparison to control group. The present study revealed that various fractions of *C. opaca* leaves ameliorated the toxic effects of CCl_4_ near to control by elevating the activity of suppressed antioxidant enzymes and reducing the level lipid peroxidation and hydrogen peroxide. However, fractions alone confirmed non significant alteration as compared to control group.

**Table 2:** **Effects of various fractions of *C*. *opaca *** **leaves on tissue proteins and antioxidant enzyme levels**

**Group**	**CAT (U/min)**	**POD (U/min)**	**SOD (U/mg protein)**	**TBARS (nM/min/mg protein)**	**H**_**2**_**O**_**2 **_**(nM/min/mg tissue)**
Control	4.21 ± 0.19c	9.05 ± 0.30c	3.00 ± 0.27c	2.87 ± 0.36c	1.432 ± 0.011e
Oil + DMSO	4.13 ± 0.26c	8.84 ± 0.27c	3.12 ± 0.21c	2.67 ± 0.33c	1.480 ± 0.016e
CCl_4_	2.47 ± 0.34a	5.03 ± 0.71a	1.05 ± 0.10a	4.11 ± 0.08a	2.524 ± 0.077a
Sily + CCl_4_	3.66 ± 0.20b	8.24 ± 0.78c	2.63 ± 0.14b	3.47 ± 0.14b	1.548 ± 0.027d
HLC + CCl_4_	3.34 ± 0.16b	5.56 ± 0.39a	1.11 ± 0.23a	4.03 ± 0.13a	2.272 ± 0.078b
ELC + CCl_4_	3.32 ± 0.22b	7.80 ± 0.68b	1.95 ± 0.27b	3.36 ± 0.06b	1.744 ± 0.072c
MLC + CCl_4_	3.75 ± 0.15b	8.07 ± 0.52b	2.21 ± 0.36b	3.32 ± 0.10b	1.617 ± 0.092c

Values are Mean ± SD (06 number). Sily = Silymarin.

a-f (Means with different letters) indicate significance at p < 0.05.

In fact, the coordinate action of various antioxidants is important so, phase II metabolizing enzymes play a vital role in for detoxification of free radicals. Table 3 illustrates the effects of various fractions of *C. opaca* leaves on GST, GPx, GR, GSH, QR and DNA fragmentation% in myocardial tissue. Chronic treatment of CCl_4_ showed a drastic (p < 0.05) decline in the level of GST, GPx, GR, GSH and QR which was restored to normal level by post-treatment of different fractions of *C. opaca* leaves. Further, DNA fragmentation% was noticeably increased (p < 0.05) due to CCl_4_ intoxication. Post-administration of different fractions of *C. opaca* leaves reversed the level of DNA fragmentation% near to control group. The results indicate that ELC or MLC administered with CCl_4_ completely suppressed the affect of CCl_4_ and increased the level of glutathione group while lessened the fragmentation level of DNA. The group of rats administered with the fractions of *C. opaca* leaves alone showed non significant alterations and were related to the control group. In case of antioxidant status, the ranking order of fractions of *C. opaca* leaves was ELC > MLC > BLC > CLC > HLC > ALC.

**Table 3:** **Effects of various fractions of *C*. *opaca *** **leaves on phase II antioxidant enzymes and DNA fragmentation**

**Group**	**GST (nM/min/mg protein)**	**GPx (nM/min/mg protein)**	**GR (nM/min/mg protein)**	**GSH (μM/g tissue)**	**QR (nM/min/mg protein)**	**DNA damages %**
Control	146.34 ± 5.62d	100.5 ± 4.38c	164.37 ± 6.31e	18.15 ± 1.36c	97.70 ± 4.12d	10.47 ± 2.46d
Oil + DMSO	139.19 ± 4.17d	105.91 ± 4.72c	158.81 ± 5.96e	19.04 ± 1.50c	102.13 ± 3.26d	11.23 ± 3.94d
CCl_4_	105.50 ± 4.23a	78.64 ± 2.38a	104.33 ± 3.33a	11.26 ± 0.65a	63.30 ± 2.36a	43.37 ± 3.66a
Sily + CCl_4_	130.53 ± 3.13c	90.56 ± 1.81b	147.18 ± 2.49d	15.28 ± 1.40b	89.14 ± 2.71c	16.40 ± 1.34c
HLC + CCl_4_	105.48 ± 2.14a	78.76 ± 1.06a	110.43 ± 3.65a	13.17 ± 0.47b	65.09 ± 2.18a	32.30 ± 3.71b
ELC + CCl_4_	114.73 ± 2.94b	84.06 ± 1.47b	133.23 ± 3.62d	14.25 ± 0.47b	75.14 ± 2.21b	15.22 ± 3.23c
MLC + CCl_4_	122.08 ± 3.49c	87.09 ± 2.02b	142.51 ± 2.20d	14.17 ± 1.16b	80.42 ± 4.08b	14.17 ± 3.02c

Values are Mean ± SD (06 number). Sily = Silymarin.

a-e (Means with different letters) indicate significance at p < 0.05.

### Effects of *C. opaca* leaves on DNA damages (ladder assay)

**Figure 1:**
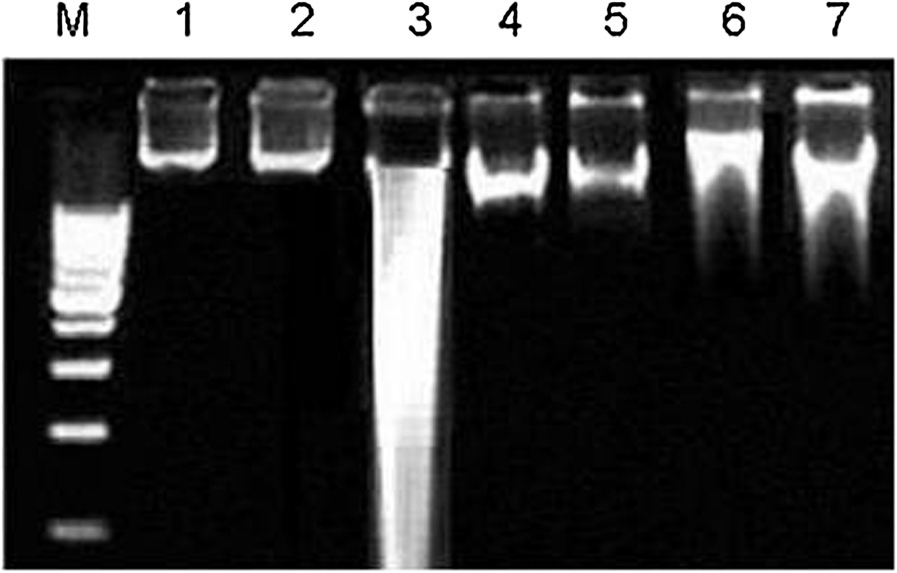
**Agarose gel showing DNA damage by CCl4 and protective effects of various fractions of C. opaca leaves in cardiac tissue.** Lanes from left (M) low molecular weight marker, (1) control, (2) DMSO + Olive oil group, (3) CCl4 group, (4) Silymarin + CCl4 group, (5) MLC + CCl4 group, (6) ELC + CCl4 group, (7) HLC + CCl4 group.

DNA was extracted from the cardiac tissue of the treated rats and the banding pattern was observed in Figure 1. Treatment of rats with CCl_4_ increased the cardiac tissue DNA damages than those of control group. A different banding pattern was observed in case of CCl_4_ treated rats that was absent from the cardiac tissues of control rats. DNA extracted from the group treated with various fractions of *C. opaca* leaves administered with CCl_4_ showed a markedly repaired DNA. The group treated with only fractions did not show any kind of DNA damages.

### Effects of *C. opaca* leaves on cardiac histoarchitecture

**Figure 2:**
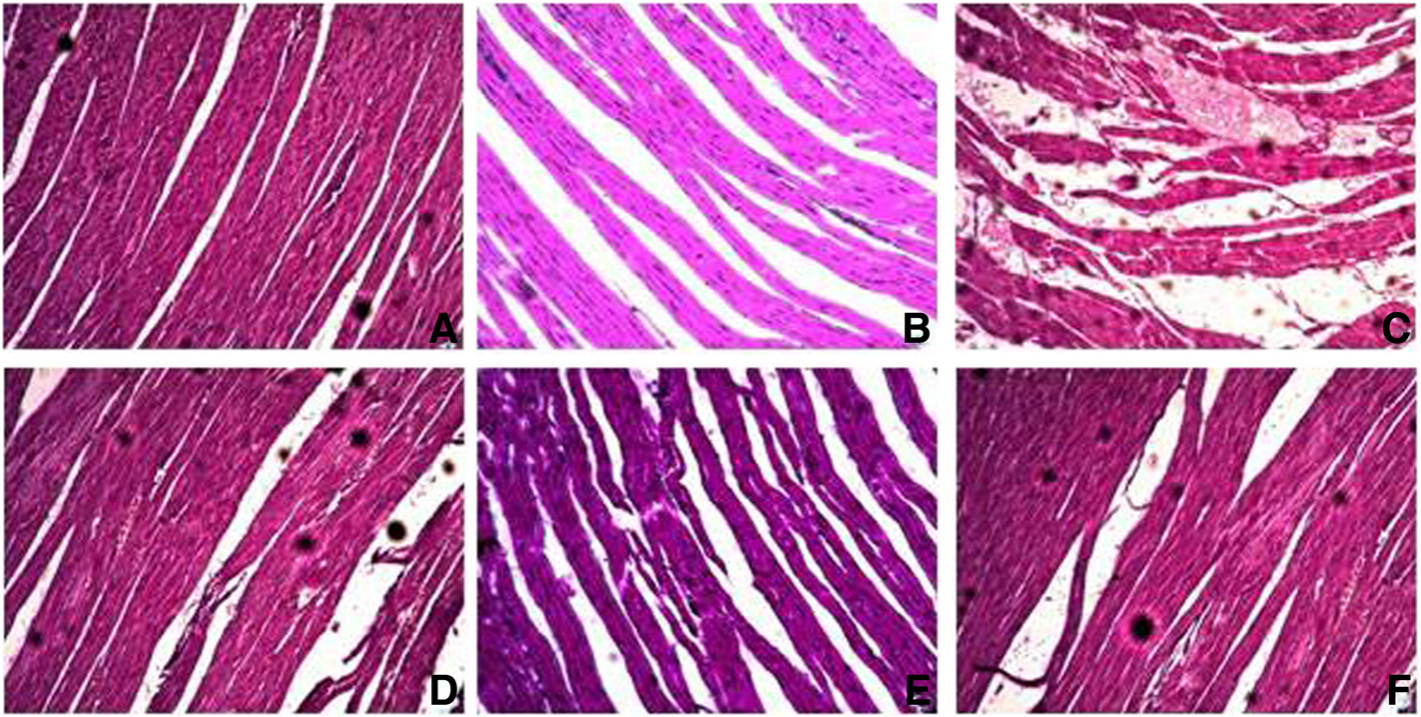
Microphotograph of rat heart (H & E stain) (A) Cardiac representative section of various groups showing normal histology, (B) DMSO + Olive oil group, (C) CCl4 group, (D) MLC + CCl4 group, (E) ELC + CCl4 group, (F) HLC + CCl4 group.

The cardiac sections of different treated groups showed differences. The histological architecture of heart from different experimental groups showed series of variations from no damage (control group) to mild lesions (extract treated group) to highly severe (CCl_4_ group). Control and DMSO groups showed normal cardiac histology of rat as represented by Figure 2A and B, respectively. In these groups heart tissue showed a normal myofibrillar structure with striations, branched appearances and continuity with adjacent myofibrils. Cardiac muscle displays several small blood vessels and capillaries in the connective tissue. The heart section of CCl_4_ treated rats showed distinctive appearance of cardiotoxicity with various degrees of focal damages, distortion in blood capillaries, and intrusion in the trabeculae of heart and retrogressive lesions in muscle fibres. Additionally, hyaline necrosis and appropriate mucoid edema with vacuolar changes were noticed in CCl_4_ treated rats as illustrated in Figure 2C. Post-treatment with different fractions of *C. opaca* leaves administered with CCl_4_ confirms its constructive effects by less capillary dilatation and vacuolar changes and leukocyte infiltration in comparison to compared to that of CCl_4_ treated groups (Figure 2d-F).

## Discussion

In the literature it has been studied that CCl_4_ can generate oxidative stress in tissues other than liver, such as kidneys, heart, lung, testis, brain and blood [27,28]. Thus, oxidative insults induced by CCl_4_ resulted in degenerative processes of various tissues. As cardiac tissue has affinity for CCl_4_ due to cytochrome P450. So, oxidative damage to lipids and proteins of heart tissues probably occurred due to CCl_4_ intoxication. In view of that, natural resources are being soughed for their potential tissue protective effects. An approach for detection of cardiac injury, tissue ischemia and myocardial infarction involves measurements of the well known cardiac marker enzymes for example, creatine kinase (CK), cardiac creatine kinase-MB fraction (CK-MB), AST, ALT, ALP, LDH, and cholesterol in serum [29,30]. The important point is that all of the above discussed enzymes are not restricted to cardiac tissue except CK and CKMB, their increased activity in serum is responsible for in non-cardiac tissue injuries like liver. CKMB, a myocardial enzyme determines the degree of myocyte injury that’s why WHO accepted it as gold standard indicator of myocardial injury [31]. The integrity of cardiac cell membrane gets disturbed as a consequence of peroxidation of membrane by oxygen-derived free radicals [32] causing leakage of enzymes. This accounts for the decreased activities of these enzymes in heart tissue because these enzymes enter into the plasma thus increasing their concentration in the serum as an indicator of myocyte injury [33]. CCl_4_ intoxication was responsible for excess release of CK and CKMB in the serum of rats and this study correlates with the commonly reported study that Dox-induced free radical generation activates cardiac myocytes disruption and peroxidation of membrane, which boosted the CKMB level of serum [34,35]. A marked reduction in the levels of CK and CK-MB, being marker parameters of heart damage in experimental groups treated with various fractions of tested samples proves improved cardiac function in CCl_4_-treated group. These serological studies have a superb correlation with histological examination of cardiac tissue of rats.

There is considerable evidence that induction of CAT, POD, SOD and phase-II detoxifying enzymes, including GPx, GR and GST can adjust the verge for chemical carcinogenesis by increasing resistance against toxic substances. Cellular antioxidant enzymes like SOD, GPx, CAT and GST are important cellular guards due to detoxifying ability against free radicals. Numerous diverse results have been reported for the variation of these antioxidant enzyme activities in rat model against CCl_4_ challenges [36]. In cellular defense system, GSH has the conjugating aptitude with metabolites/free radicals, thus stabilizing the membranes from detrimental effects of lipid peroxides. Depletion in GSH content indicates the condition of oxidative stress caused by adriamycin administration [37]. It also has been reported that cellular GSH depletion is closely related with lipid peroxidation induced by toxic agents [38]. Lipid peroxidation is also involved in pathogenesis of adriamycin-induced cardiomyopathy. The present remarks are in conformity with previous reports, which showed that myocardial antioxidant defense mechanism was working at a lower rate regardless of higher degree of oxidative trauma in CCl_4_-induced cardiomyopathy state. In the present investigation, rats treated with various fractions of tested samples practiced abridged lipid peroxidation with enhanced GSH level and GPx, GST and GR activities. It appears therefore that various fractions of tested samples protects the cardiac tissue against CCl_4_-induced lipid peroxidation and has the antioxidant propensity.

ROS have an immense damage to DNA, causing DNA mutations responsible for various degenerative diseases. Several specific and unspecific repair enzymes remove oxidative DNA modifications. Since oxidative DNA damage is continuously induced and repaired, a steady-state level of oxidative DNA damage is expected under normal conditions. Oxidative stress causes an increase in oxidative DNA damage [39]. Regarding to present findings CCl_4_ cause the degradation of DNA by generating free radicals. Similar finding have also been described by Manierea *et al*. [40] that the chemicals such as CCl_4_, and other xenobiotic compounds induce the production of reactive free radicals which cause the oxidative damage to DNA, with formation of DNA adducts and genetic mutations. The DNA damage in various tissues like brain, testis, adrenal and liver was reported but data for DNA damage is scanty for cardiac tissue. Administration of various fractions of tested samples in experimental groups to CCl_4_ intoxicated rats confined the cardiac tissues and there was a marked decline in percentage of fragmented DNA that was further confirmed by DNA ladder assay.

The microscopic structural changes in the heart tissues of CCl_4_ intoxicated rats had similarity with previous report of Jayakumar *et al*. [41]. Co-treatment with various fractions of tested samples in experimental groups showed substantial avoidance in the structural alterations. This indicates that administration of various fractions of tested samples scavenged the free radicals to stop cellular damages. Previous reports have also confirmed the beneficial effects of supplementation of antioxidants in adriamycin induced myocardial injury. Histopatholgical studies revealed myocardial atrophy, nuclear pyknosis, and cytoplasmic vacuoles in the CCl_4_ treated hearts. Similar observations have also been made in earlier studies on acute doxorubicin induced cardiotoxicity [42,43]. Regarding the comparative studies of CCl_4_ with other toxic chemicals on cardiac tissue, similar toxic effects were reported by with other chemicals [44]. In the present study the main effects of CCl_4_ was the prominent fatty change congestion in the blood vessel clearing of cytoplasm with foamy appearance and nuclear degeneration in some area was observed which was significantly recovered by various fractions of tested samples that may be attributed to the individuals or combined action of bioactive compounds present in respective fraction. Similar results were reported by the study of Khan *et al*. [45] and Eshaghi *et al.*[46] extract ameliorated the CCl_4_-induced cardiotoxicity of male albino rats.

## Conclusion

The protective effect observed in this study provide some mechanistic evidence for why indigenous people of Pakistan and other Asian countries found it useful for the cariadic ailments as well as food additive.

## Competing interest

The authors declare that they have no competing interests.

## Authors’ contributions

SS made significant contribution to acquisition and interpretation of data, conception and drafting of the manuscript. MRK, HMA and RAK (ORCID ID: 0000-0003-0453-2090) has made substantial contribution to conception and design, interpretation of data and drafting for intellectual content. All authors read and approved the final manuscript.
